# ““Why shouldn’t I expect things from life?” – what people with lived experience from psychosis highlight as important to their personally defined long-term recovery process”

**DOI:** 10.1192/j.eurpsy.2023.315

**Published:** 2023-07-19

**Authors:** G. Åsbø, H. Haavind, S. Hembre Kruse, K. Fjelnseth Wold, W. Ten Velden Hegelstad, K. Lie Romm, T. Ueland, I. Melle, C. Simonsen

**Affiliations:** 1 NORMENT, Division of Mental Health and Addiction, Oslo University Hospital and Institute of Clinical Medicine, University of Oslo, Norway; 2 Early Intervention in Psychosis Advisory Unit for South-East Norway, Division of Mental Health and Addiction, Oslo University Hospital, Norway; 3 University of Oslo; 4 TIPS Centre for Clinical Research in Psychosis, Stavanger University Hospital, Stavanger, Norway, Oslo, Norway

## Abstract

**Introduction:**

Many people with lived experience from psychosis recover and thrive, contrary to the common stigmatizing belief that they will be chronic “patients”. But there are several ways to understand recovery, one is as a subjective process best explored through qualitative interviews with people who have recovered from psychosis. However, there is a need for more qualitative interview studies exploring what has been important for long-term subjective recovery for people with lived experience from psychosis outside of treatment. Exploring themes that are novel than previous research will have important clinical implications.

**Objectives:**

This study aims to qualitatively explore what people with lived experience from psychosis believe has been the most important to attain and sustain their long-term personally defined recovery.

**Methods:**

Qualitative interviews with 20 individuals participating in two follow-up-studies (TOP and TIPS-study) 10 and years 20 years after first treatment for a psychotic disorder (schizophrenia- or bipolar spectrum), respectively. All participants were in either clinical recovery (symptom remission and adequate functioning) or personal recovery (self-rated questionnaire) or both. Interviews were analyzed with thematic analysis in group meetings between the PhD-candidate, the main supervisor, a professor emerita in qualitative method and a co-researcher with lived experience from bipolar disorder.

**Results:**

Participants defined recovery differently, but: “understanding myself”, “stable symptoms” and “finding the life that is right for you” were of the most common definitions. Tentatively, five main themes appear to be the most salient contributions to recovery: 1. Balance stress management with taking risks and following personal goals. 2. Accepting experience/”owning your story” in order to strategically disclose and manage stigma. 3. Taking agency over own recovery and mastery of everyday life. 4. Social support is crucial, but should change over time depending on need. 5. Feeling a sense of belonging to society does not need to entail “normality”.

**Conclusions:**

Recovery was defined differently by each participant, but common themes across participants highlight that appropriate risk-taking, accepting your experience/owning your story, sense of agency, social support and inclusion are important to long-term recovery in psychosis.

**Disclosure of Interest:**

None Declared

